# Design of Mucosal Vaccines Against Swine Enteric Coronaviruses: From Antigen Delivery to Immune Activation

**DOI:** 10.1155/tbed/3230453

**Published:** 2025-11-23

**Authors:** Qihao Pan, Yinhe Sun, Haojie Bai, Wenqian Wang, Borui Liu, Muzi Li, Ao Gao, DongFang Zheng, Weili Jiang, Hui Hu, Honglei Zhang, Yuqiang Xiang, Zhanyong Wei, Lanlan Zheng

**Affiliations:** ^1^College of Veterinary Medicine, Henan Agricultural University, Zhengzhou 450046, P. R., China; ^2^Ministry of Education Key Laboratory for Animal Pathogens and Biosafety, Zhengzhou 450046, P. R., China; ^3^Henan Province Key Laboratory for Animal Food Pathogens Surveillance, Zhengzhou 450046, P. R., China; ^4^Longhu Laboratory of Henan Province, Zhengzhou 450046, P. R., China

**Keywords:** mucosal immunity, SeCoVs, vaccine

## Abstract

Swine enteric coronaviruses (SeCoVs) cause acute enteritis and high mortality in neonatal piglets, posing a significant threat to the swine industry. Injectable vaccines often fail to induce effective mucosal immunity, and their efficacy is further compromised by maternally derived antibodies. Oral and intranasal mucosal vaccines offer promising alternatives, enabling localized and durable protection. This review summarizes recent advances in mucosal vaccines against SeCoVs, focusing on antigen delivery platforms and mucosal immune activation. Novel antigen delivery platforms, including nanoparticles (NPs), hydrogels, engineered probiotics, recombinant viral vectors, and eukaryotic expression systems, have improved antigen stability and facilitated transport across the epithelium to mucosal inductive sites. Moreover, targeting strategies that focus on microfold cells (M cells) and dendritic cells (DCs) enhance antigen uptake and presentation. These delivery systems promote mucosal immune activation by inducing secretory IgA (sIgA), maintaining Th1/Th2 balance, and promoting the generation of T and B cells. In addition, the incorporation of adjuvants further strengthens these responses, resulting in more robust and durable protection. By synergistically integrating advanced mucosal vaccine delivery systems with rational adjuvant strategies, this review provides theoretical and practical perspectives for the development of safe, effective, and broadly protective mucosal vaccines targeting SeCoVs infections.

## 1. Introduction

Coronaviruses are enveloped, single-stranded, and positive-sense RNA viruses. Swine enteric coronaviruses (SeCoVs) mainly include porcine epidemic diarrhea virus (PEDV), porcine deltacoronavirus (PDCoV), transmissible gastroenteritis virus (TGEV), and swine enteric alphacoronavirus (SeACoV). Among these, PEDV and PDCoV being the most important threats to global swine health [1–3]. SeCoVs are usually transmitted through the oral route, infecting intestinal villous epithelial cells and causing severe gastrointestinal symptoms, such as watery diarrhea, vomiting, and dehydration, which often result in high mortality in suckling piglets [4]. Since 2010, variant PEDV strains have experienced frequent outbreaks across many parts of Asia, resulting in serious economic losses [5–7]. PDCoV, first reported in North America and subsequently in several Asian countries since 2014, mortality in neonatal piglets can exceed 40% [8–10]. TGEV, initially identified in the United States in 1946, is characterized by acute gastroenteritis with up to 100% mortality in suckling piglets [11–13]. SeACoV, a novel *alphacoronavirus* phylogenetically linked to bat-origin HKU2-like viruses, emerged in Guangdong Province of China in 2017, triggering a widespread outbreak of fatal diarrheal disease in piglets and demonstrating a high propensity for inducing severe intestinal pathology [14].

Currently licensed vaccines against SeCoVs are mainly based on inactivated or live attenuated formulations administered intramuscularly, which are designed to induce systemic IgG responses. However, the efficacy of these injectable vaccines is considerably limited especially in neonatal piglets, primarily due to their immature immune systems and inherently weak capacity for mounting robust humoral and cellular immune responses, which consequently results in inadequate protective immunity in suckling piglets [15]. Additionally, maternally derived IgG antibodies that are transferred through colostrum can interfere with immunity induced by vaccines and reduce its effectiveness [16]. Intramuscular vaccines also do not effectively stimulate gut-associated lymphoid tissue (GALT) or mucosal immune response, and therefore, fail to induce protective secretory IgA (sIgA) [17]. In the mucosal immune system, sIgA plays a key role in establishing the primary mucosal immune barrier, maintaining intestinal homeostasis, and preventing viral spread and disease progression [18, 19]. Oral and intranasal mucosal vaccines have demonstrated distinct advantages that they directly engage mucosal defense mechanisms while simultaneously eliciting both local and systemic immune responses. This review summarizes recent advances in antigen delivery platforms and immune activation strategies for mucosal vaccines against SeCoVs. Furthermore, we discuss the optimization of targeted delivery, trans-epithelial antigen uptake, and adjuvant design to enhance vaccine efficacy, thereby providing valuable insights for the prevention and control of SeCoVs infections.

## 2. SeCoVs Infection and Intestinal Mucosal Immunity

SeCoVs cause infection primarily through the intestinal tract, where viral replication occurs in specialized epithelial and immune cells. The structure and function of the porcine intestinal mucosal immune system critically influence both viral pathogenesis and the efficacy of protective immunity. Understanding infection routes, the key components of intestinal mucosal defense, and the immune responses induced by mucosal vaccination provides the basis for developing effective strategies against SeCoVs.

### 2.1. SeCoVs Infection Routes and Target Cells

SeCoVs are highly contagious and environmentally stable pathogens that primarily transmit via the fecal-oral route. Moreover, epidemiological studies have demonstrated that indirect transmission through contaminated airborne, transport vehicles, feed, boots, and farm personnel contributes to the spread of infection during outbreaks [20]. Field investigations have further confirmed that lactogenic transmission of PEDV and TGEV occurs in sows, establishing maternal milk as an additional infection route [21, 22]. After entering the host, SeCoVs preferentially replicate in the intestinal epithelium, with villus enterocytes (villin^+^) in the small intestine serving as the principal cellular targets. Studies using porcine intestinal enteroid models have revealed that PEDV is also capable of infecting intestinal stem cells (LGR5^+^) and goblet cells (MUC2^+^) [23–25]. PDCoV infects Sox9^+^ stem cells and villin^+^ enterocytes, triggering activation of the Notch signaling pathway, which subsequently suppresses goblet cell differentiation and decreases mucus secretion [26, 27]. Although direct experimental evidence of SeCoVs infection in microfold cells (M cells) remains limited, these epithelial portals for luminal antigens play a pivotal role in initiating mucosal immune responses.

### 2.2. Components of the Porcine Intestinal Mucosal Immune System

**Figure 1:**
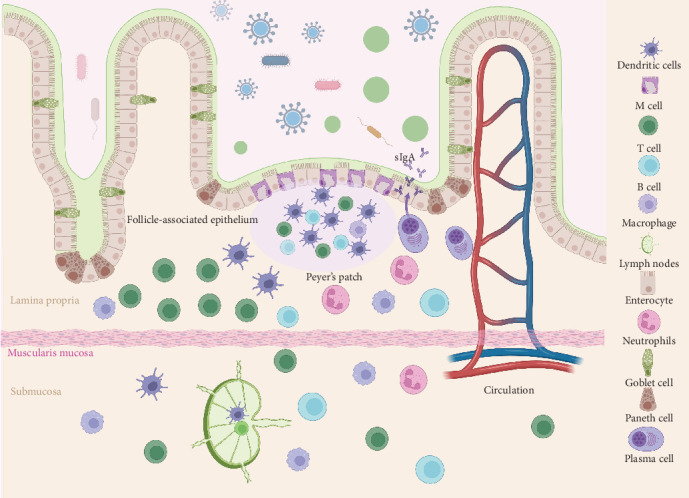
Organization of the porcine intestinal mucosal immune system. The intestinal mucosa contains villi for nutrient absorption and PPs as major inductive sites of GALT. The FAE covering PPs includes specialized M cells that transport luminal antigens to DCs and macrophages in the subepithelial dome. Within PPs, antigen presentation activates T and B lymphocytes, initiating GC reactions that drive B cell differentiation. Plasma cells generated in this process migrate to the lamina propria and sIgA which is released into the lumen to neutralize pathogens and block epithelial adhesion.

The porcine intestinal mucosal immune system is composed of specialized immune cells and organized lymphoid tissues that function as the primary barrier against enteric viral infection. As illustrated in Figure 1, Peyer's patches (PPs), M cells, dendritic cells (DCs), and plasma cells constitute the core elements that mediate antigen uptake, presentation, and sIgA production.

PPs, which are major components of the GALT, are primarily found in the ileum and consist of multiple lymphoid follicles that contain dense populations of B lymphocytes, T lymphocytes, DCs, and macrophages. The follicles are covered by the follicle-associated epithelium (FAE), which contains specialized M cells [28]. M cells are characterized by a thin glycocalyx, sparse microvilli, and high expression of receptors such as glycoprotein 2 (GP2) and *β*1 integrins. These features enable them to efficiently internalize luminal antigens through endocytosis or micropinocytosis and deliver them to the underlying antigen-presenting cells (APCs), particularly DCs and macrophages [29, 30]. DCs are professional APCs distributed throughout mucosal tissues, including the skin, respiratory tract, gastrointestinal tract, and urogenital tract [31]. Within PPs, DCs capture, process, and present antigens delivered by M cells to naïve T lymphocytes, thereby initiating adaptive immune responses [32, 33]. The antigen-specific activation of T cells provides essential help to B lymphocytes, promoting their proliferation and differentiation [34]. Activated B cells ultimately differentiate into plasma cells that migrate to the lamina propria and become the primary source of sIgA in mucosal immunity [35]. The sIgA antibodies produced by plasma cells bind to pathogens in the gastrointestinal and respiratory tracts, thereby preventing microbial adhesion and penetration of epithelial cells and establishing a crucial line of protection against infection [36–38]. Together, these sequential processes of antigen uptake by M cells, antigen presentation by DCs, and B cell activation and differentiation into sIgA-producing plasma cells define the central immunological function of PPs in mucosal protection.

### 2.3. Major Immune Responses Induced by Mucosal Vaccines

**Figure 2:**
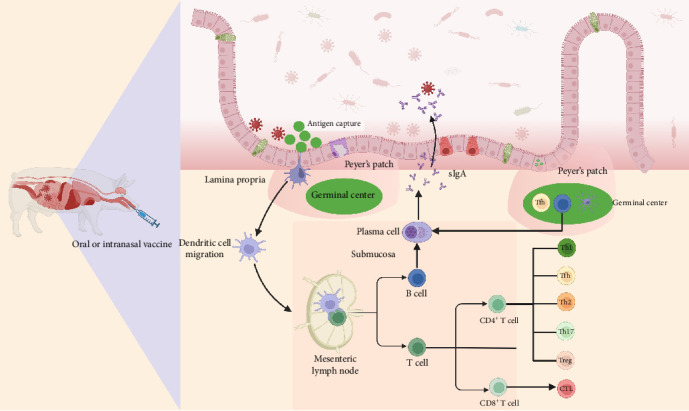
Process of intestinal mucosal immune activation induced by oral or intranasal vaccines. Following mucosal vaccination, antigens are transcytosed by M cells in the FAE and captured by DCs and macrophages in the subepithelial dome of PPs. DCs migrate to inductive sites, including the MLN, where they activate naïve CD4^+^ and CD8^+^ T cells through MHC class II and MHC class I presentation, respectively. Activated CD4^+^ T cells differentiate into subsets, such as Th1, Th2, Th17, Treg, and Tfh cells. Tfh cells, together with FDCs, support GC reactions that promote B cell proliferation, affinity maturation, and IgA class switching. Activated B cells migrate to the lamina propria, where they differentiate into plasma cells that secrete dimeric IgA. sIgA is transported into the intestinal lumen via the polymeric Ig receptor (pIgR), where it neutralizes pathogens and prevents epithelial adhesion. CD8^+^ T cells and T_RM_ provide additional cytotoxic and cytokine mediated protection.

Mucosal vaccination differs fundamentally from injectable vaccines by eliciting sIgA and tissue-resident memory T cells (T_RM_), which intercept pathogens at epithelial barriers and provide immediate protection at the earliest stage [19]. As illustrated in Figure 2, antigens taken up by M cells are processed by DCs and presented within mucosa-associated lymphoid tissue (MALT), where the cooperation of T helper (Th) cells and mucosa-specific cytokines promotes IgA class switching and the differentiation of IgA^+^ plasma cells [39, 40]. These plasma cells migrate to the lamina propria and sustain continuous secretion of dimeric sIgA, which neutralizes pathogens, prevents epithelial adhesion, and contributes to mucosal homeostasis [41]. Besides, B cell responses undergo further optimization in the germinal centers (GCs) of PPs and nasal-associated lymphoid tissue (NALT), where T follicular helper (Tfh) cells and follicular DCs (FDCs) regulate clonal expansion, affinity maturation, and class-switch recombination [42–45]. This GC activity ensures the generation of high-affinity, long-lived IgA responses that provide durable mucosal protection and lay the foundation for long-term humoral immunity [46].

T cell immunity is an essential component of mucosal vaccine induced protection. After antigen presentation by DCs, naïve CD4^+^ T cells differentiate into Th1 and Th2 subsets that respectively promote antiviral cytotoxicity and IgA-mediated humoral responses, while maintaining an essential functional balance [47, 48]. Mucosal vaccination also induces durable memory T cell populations, including effector memory T cells (T_EM_), central memory T cells (T_CM_), and T_RM_ [49, 50]. T_RM_ play a pivotal role at mucosal barriers including the intestinal epithelium and respiratory tract by maintaining long-term persistence, secreting effector cytokines, including IL-2 and interferon (IFN)-*γ*, and mounting rapid effector responses upon pathogen re-exposure independent of antigen representation [51, 52]. Compared with injectable vaccination, which predominantly induces systemic T_CM_ responses, mucosal vaccination preferentially promotes T_RM_ and T_EM_, thereby establishing more effective frontline defense against enteric and respiratory viral infections and highlighting the importance of localized T cell memory in protective mucosal immunity [53, 54].

## 3. Antigen Delivery Platforms for Oral and Intranasal Mucosal Vaccines

The development of effective oral and intranasal mucosal vaccines requires antigen delivery systems that maintain antigen stability in hostile environments and enable efficient engagement of mucosal immune inductive sites. Current research has focused on several representative strategies, including nanoparticle (NP)-based carriers, hydrogels, engineered probiotics, recombinant viral vectors, and eukaryotic expression systems, each providing distinct advantages for overcoming the barriers of mucosal vaccination. A comparison of structural features, immune activation mechanisms, and representative applications of oral and intranasal mucosal vaccine delivery platforms is presented in Table 1.

### 3.1. NPs

Nanomaterials have emerged as versatile platforms for mucosal vaccine delivery due to their tunable particle size, surface modifiability, and favorable biocompatibility [65]. Among them, NP based systems are particularly advantageous, as they protect antigens from degradation by gastric acid and digestive enzymes while facilitating trans-epithelial transport, thereby enhancing uptake by M cells and subsequent activation of DCs [66, 67]. Representative NP formulations include natural polymers, such as chitosan and alginate; synthetic polymers, such as poly (lactic-co-glycolic acid) (PLGA); lipid-based carriers, such as lipid NPs (LNPs); inorganic carriers.

#### 3.1.1. Chitosan

Chitosan, a cationic natural polysaccharide, has been widely applied in oral and intranasal mucosal vaccines as chitosan exhibits biocompatibility, biodegradability, and surface modifiability, while also providing intrinsic adjuvant activity [68]. The positive charge of chitosan enables strong electrostatic binding with the negatively charged mucosal epithelium, which not only improves adhesion and prolongs antigen retention at administration sites but also enhances antigen uptake by M cells and DCs, thereby strengthening local immune responses [69]. Oral administration of inactivated PEDV encapsulated within alginate-chitosan microcapsules demonstrated that encapsulation protects antigens from enzymatic degradation, facilitates antigen delivery to intestinal inductive sites, and induces elevated mucosal sIgA responses together with systemic IgG and neutralizing antibody production [70]. However, despite their excellent mucoadhesive and biocompatible properties, chitosan-based systems may exhibit limited mechanical stability and control over antigen release. These limitations have driven increasing interest in synthetic polymeric carriers, such as PLGA, which allow more precise tuning of degradation rates and immune activation kinetics.

#### 3.1.2. PLGA

Synthetic polymers represented by PLGA have been extensively studied as carriers for mucosal vaccines. PLGA NPs are characterized by high antigen encapsulation efficiency, tunable release profiles, and safety record supported by Food and Drug Administration (FDA) approval for biomedical applications [71]. Antigen encapsulation within PLGA matrices confers protection against gastrointestinal degradation while enabling controlled release in the intestinal environment, which creates conditions favorable for sustained immune stimulation [72]. The physicochemical properties of PLGA NPs, including particle size and surface modifications, determine the efficiency of translocation across M cells and targeting of inductive sites, such as PPs and mesenteric lymph nodes (MLN), where enhanced sIgA secretion, elevated IgG production, and improved memory T cell responses are observed [73]. Oral delivery of inactivated PEDV encapsulated in PLGA NPs formulated with ginseng stem-leaf saponins (GSLS) as an adjuvant demonstrated enhanced DC activation, increased neutralizing antibody titers, robust sIgA responses, and improved memory T cell formation in mice [55]. Intranasal immunization of pregnant sows with a PLGA-encapsulated inactivated PEDV vaccine induced higher PEDV-specific IgG and IgA responses and provided passive protection to neonatal piglets, resulting in reduced morbidity and mortality compared with conventional vaccination [74]. PLGA NPs ensure controlled antigen release and potent immune activation; however, limitations in encapsulating fragile biomolecules such as mRNA and proteins have driven the development of LNP systems that enable stable and efficient antigen delivery.

#### 3.1.3. LNPs

LNPs, composed of ionizable lipids, cholesterol, phospholipids, and PEG-lipids, have become the leading platform for mRNA vaccine delivery, as their structural components collectively provide high antigen encapsulation efficiency, potent membrane fusion ability, and favorable biodegradability [75, 76]. Optimized LNPs with controlled lipid composition, particle size, and surface charge can cross mucosal barriers, protect antigens from degradation, and promote efficient uptake by APCs, making LNPs suitable for oral and intranasal vaccination [77, 78]. An intranasal mRNA-LNP vaccine encoding the SARS-CoV-2 S protein induced systemic IgG together with mucosal IgA responses in hamsters, which reduced viral loads and mitigated lung pathology [57]. Similarly, a glycyrrhizin acid-based LNP system delivering the PEDV N-terminal domain antigen elicited robust antibody production and T cell activation in mice [56]. Although LNPs offer superior encapsulation and transfection efficiency, their rapid clearance from mucosal surfaces can restrict antigen exposure time. Consequently, hydrogel-based delivery systems have been designed to prolong antigen residence and provide controlled, sustained immune stimulation.

### 3.2. Hydrogel Systems

Hydrogel systems have attracted increasing attention as carriers for mucosal vaccines as the three-dimensional crosslinked polymer networks, characterized by high water content and favorable tissue compatibility, provide a supportive matrix for antigen stabilization and delivery [79, 80]. The tunable mechanical properties of hydrogels, together with their responsiveness to environmental stimuli, such as temperature and pH, enable the formation of adherent layers that prolong antigen residence and reduce mucociliary clearance [81]. Bacterial nanocellulose/polyacrylic acid (BNC/PAA) hydrogel microparticles stabilized antigens and promoted sustained release, which enhanced targeting to MALT, such as NALT and PPs, resulting in elevated IgG and sIgA responses [81]. Similarly, a hydrogel constructed from natural small molecules and metal ions exhibited strong adhesion and temperature sensitivity, and intranasal administration of this formulation prolonged antigen retention, improved DC maturation, and activated APCs in NALT, ultimately inducing both mucosal IgA and T cell responses [82]. In addition, a thermosensitive chitosan hydrogel loaded with influenza peptide antigens further demonstrated that sustained antigen presence in the upper respiratory tract facilitated the establishment of nasal-resident T_RM_ CD8^+^ T cells [83]. Moreover, a calcium-chitosan-alginate hydrogel encapsulating PEDV, administered orally or intranasally in mice, induced increases in intestinal and tear sIgA, enhanced serum IgG levels, and elevated IFN-*γ* concentrations, confirming that hydrogel-based carriers can stabilize antigens, extend mucosal residence, and induce both mucosal and systemic immune responses [58]. Beyond synthetic and polymeric matrices, biologically derived carriers have gained significant interest for their ability to colonize mucosal niches and interact directly with the host immune system. Engineered probiotic vectors represent a living delivery strategy capable of continuous antigen expression at mucosal sites.

### 3.3. Engineered Probiotic Carriers

Engineered probiotics, particularly lactic acid bacteria, such as *Lactobacillus casei* and *Lactococcus lactis*, have become prominent vectors for oral and intranasal mucosal vaccines since their capacity to survive in the gastrointestinal tract, inherent immunomodulatory activity, and Generally Recognized as Safe (GRAS) status collectively provide a strong foundation for vaccine delivery [84]. Engineered probiotics colonize mucosal surfaces and sustain antigen expression, which enables prolonged contact with APCs, such as M cells and DCs and promotes efficient induction of mucosal and systemic immunity. Studies demonstrate that oral or intranasal delivery of probiotic-based vaccines elicits sIgA production at mucosal sites and also generates robust systemic IgG responses [85, 86]. Evidence from the COVID-19 pandemic further demonstrated that probiotic supplementation upregulated antiviral gene expression in the gut and attenuated inflammation, highlighting additional potential of engineered probiotic carriers in the design of respiratory virus vaccines [87]. A recombinant *Lactobacillus* strain co-expressing an M cell-targeting peptide (Co1) and a DC-targeting peptide (DCpep) induced significantly higher serum IgG, intestinal sIgA, and Th2-type cellular responses after oral immunization in mice [59]. Similarly, a full-length PEDV S protein expressed in *Lactococcus lactis* and encapsulated in alginate-chitosan microcapsules not only increased mucosal sIgA and serum IgG but also promoted the secretion of cytokines associated with Th1 and Th2 responses [88]. Furthermore, a recombinant *Lactobacillus casei* strain expressing conserved neutralizing epitopes from PEDV, TGEV, and PoRV elicited broad-spectrum mucosal and systemic immunity in pig, demonstrating the feasibility of developing multipathogen oral vaccines against enteric diseases in swine [61].

Despite the benefits of engineered probiotics, traditional systems often rely on antibiotic resistance genes as selection markers, which raises significant biosafety and regulatory concerns, particularly for clinical and large-scale veterinary applications. To address these issues, CRISPR-Cas systems have been developed, allowing for antibiotic-free, site-specific, and stably integrated engineered probiotic strains [89–91]. The precise insertion of antigen genes into chromosomal “safe harbor” sites, coupled with the elimination of plasmid backbones and resistance markers, results in strains with enhanced genetic stability, improved biosafety, and greater colonization capacity [92]. For instance, a CRISPR-Cas-edited antibiotic-free *Lactobacillus casei* W56 strain demonstrated the ability to induce robust mucosal and systemic neutralizing IgA and IgG responses against pseudorabies virus (PRV) in mice after oral administration, highlighting the potential of the system for SeCoVs vaccine development [60]. Engineered probiotics constitute a safe and biocompatible platform but are limited in antigen expression and delivery efficiency, whereas recombinant viral vectors enable highly efficient transgene delivery and strong immune activation through viral mimicry.

### 3.4. Recombinant Viral Vectors

Recombinant viral vector vaccines employ genetically engineered viruses to deliver and express target antigens in host cells, a mechanism that induces both humoral and cellular immune responses. Commonly investigated vectors include adenoviruses, attenuated vaccinia viruses, and baculoviruses, which can mimic natural infection pathways and efficiently activate mucosal immunity, thereby providing attractive platforms for mucosal vaccine development [93]. An intranasally administered recombinant PanAd3-MVA vector elicited robust nasal sIgA responses, activated NALT, and promoted pulmonary CD8^+^ T cell infiltration in both mice and primates, while intramuscular vaccination with the same construct showed inferior efficacy in preventing upper respiratory tract respiratory syncytial virus (RSV) infection [64]. A recombinant baculovirus expressing the influenza M2 antigen for nasal delivery generated strong pulmonary CD8^+^ T cell responses and conferred effective protection without inducing immunopathology [94]. An oral recombinant adenovirus type 5 (rAd5) vaccine expressing the PEDV S protein significantly reduced viral loads in pig and induced both neutralizing antibodies and serum IgG [62]. A rAD expressing a single-chain variable fragment (SCFV) against the PEDV nucleocapsid protein upregulated intestinal IFN-*λ* expression and inhibited viral replication after oral administration in pigs, supporting the feasibility of therapeutic mucosal vaccination [95]. Furthermore, a rAD co-expressing the PEDV S1 and N proteins (rAd5-S1-N) induced strong mucosal and systemic immune responses, enhanced IFN-*γ*-secreting T cell activity, and reduced viral shedding and intestinal pathology after intranasal or parenteral immunization in mice and pig. The same construct also provided maternal passive immunity, which underscores the value of rAd5-S1-N as a candidate for PEDV prevention [63]. Recombinant viral vectors have demonstrated strong immunogenicity and the capacity to elicit both mucosal and systemic immune responses. However, concerns regarding vector safety, genetic stability, and large-scale production have prompted the exploration of alternative systems for antigen expression, such as eukaryotic expression platforms.

### 3.5. Eukaryotic Expression Systems

Eukaryotic expression systems, such as yeast-based systems, insect cell-baculovirus expression systems (BEVS), mammalian cell systems, and plant-based expression platforms, enable the production of complex post-translational modifications that are not achievable in prokaryotic hosts. While eukaryotic expression systems are not mucosal delivery platforms as such, they provide the antigenic foundation upon which mucosal vaccine strategies against SeCoVs can be built.

BEVS has been most extensively utilized on account of its capacity to achieve high protein yields and facilitate mammalian-like glycosylation, and it has been employed to produce several licensed vaccines such as Flublok for influenza as well as commercial vaccines against human papillomavirus (HPV) and avian influenza [96, 97]. A recombinant BEVS-based system expressing the S, M, and E proteins of PDCoV successfully assembled virus-like particles (VLPs), which elicited strong IgG and neutralizing antibody responses in mice, indicating their potential for oral or intranasal administration [98]. Additionally, codon optimization of the PDCoV receptor-binding domain (RBD) and its expression in BEVS produced a purified protein that induced both humoral and cellular immune responses in mice [99]. Moreover, BEVS-based expression of S proteins from PEDV, TGEV, and PDCoV facilitated the development of a trivalent vaccine candidate, which significantly boosted IgG titers and cytokine responses in mice [100]. Yeast expression systems offer several advantages, including rapid cell growth, low production costs, and basic eukaryotic post-translational modifications, features that have supported the development of vaccines against hepatitis B, influenza, and HPV [101]. Mammalian cell systems, which remain the gold standard for recombinant protein production given their capacity to achieve authentic human-like glycosylation and their broad application in monoclonal antibody and VLP production, are often limited by high costs and extended production timelines [102]. Plant-based expression systems are particularly suited for mucosal vaccines in resource-limited settings due to their low cost, high scalability, and lack of cold-chain requirements, a combination of advantages that has already enabled several plant-derived vaccine candidates to advance to clinical evaluation [102]. By enabling the production of structurally authentic SeCoVs antigens with proper folding and glycosylation, eukaryotic expression systems lay the groundwork for optimizing targeted delivery and trans-epithelial uptake to achieve stronger mucosal immune responses.

## 4. Optimizing Targeted Delivery and Trans-Epithelial Antigen Uptake for Mucosal Vaccination

While delivery platforms establish the structural basis for antigen protection and transportation, the effectiveness of mucosal vaccines in eliciting immune responses is significantly influenced by the ability to target antigens to specific mucosal inductive sites. Successful antigen uptake relies on precise targeting of specialized epithelial cells, such as M cells and DCs, which play crucial roles in the transcytosis of antigens across mucosal barriers. Achieving efficient antigen transport across the epithelial layer, followed by precise delivery to APCs, is essential for initiating robust immune responses at mucosal surfaces.

### 4.1. M Cell and DC-Targeting Designation

M cells which are specialized epithelial cells located in MALT, translocate luminal antigens across the epithelial barrier to underlying immune cells, thereby establishing them as a central point of entry for both oral and intranasal vaccines. The primary goal of M cell-targeted delivery is to enhance transcytosis efficiency through high-affinity binding to surface receptors, thereby enhance the downstream immune responses [103]. The RGD (Arg-Gly-Asp) tripeptide and the Co1 peptide can bind to integrin *α*v*β*3 or GP2 on M cells to promote antigen uptake [104, 105]. Additionally, PEG-PLGA NPs, which are modified with RGD, exhibit strong binding to *β*1 integrin on M cells, resulting in increased antigen accumulation in PPs and elevated IgG responses in vivo [106]. Moreover, mesoporous silica NPs (MSNs) coated with chitosan-catechol and conjugated with a PEDV COE-RGD antigen have been shown to enhance intranasal mucosal uptake by promoting DC recruitment, improving lymphatic transport, and transiently altering the distribution of ZO-1 to increase epithelial permeability, thereby inducing robust cellular, humoral, and neutralizing immune responses [107]. Antigen transcytosis is initiated by M cells and followed by DC capture and processing, together ensuring efficient activation of mucosal immune responses.

DCs as professional APCs, play a crucial role in mucosal immunity and serve as a central target in vaccine design. Targeting DCs enhances antigen uptake, processing, and presentation, thus promoting robust T cell activation and the establishment of long-term immune memory. Various targeting agents have been developed to target DCs, including DCpep peptides, anti-DC-SIGN antibody fragments, and mannose-conjugated ligands. These targeting agents interact with surface receptors such as DC-SIGN, mannose receptors, and DEC205, which facilitate antigen delivery and immune activation[108, 109]. Engineering *Lactobacillus casei* to express PEDV COE fused with both Co1 and DCpep peptides enables simultaneous targeting of M cells and DCs, resulting in enhanced antigen delivery [59]. Furthermore, a study demonstrated that a *Lactobacillus casei* strain expressing a Co1-fused COE antigen significantly increased antigen uptake in PPs and elicited stronger immune responses compared to nontargeted strains after oral administration [110]. By simultaneously targeting M cells and DCs, mucosal vaccines can achieve more efficient antigen transport and presentation.

### 4.2. Enhancing Trans-Epithelial Transport and Antigen Uptake

The intestinal epithelial barrier, which is structurally supported by adherens junctions (AJs) and its permeability is primarily regulated by tight junctions (TJs), constitutes a major obstacle for oral antigen delivery [111]. One strategy to improve trans-epithelial transport is the temporary adjustment of TJs, which increases paracellular permeability and facilitates antigen entry into PPs and other mucosal lymphoid tissues [112]. NP and vesicle-based systems can modulate TJ proteins, such as ZO1 and claudin1, thereby facilitating antigen transport across the epithelial barrier and enhancing mucosal immune activation [80]. Another natural pathway that contributes to antigen uptake involves DC capture, in which DCs extend dendrites through epithelial gaps near M cells to directly sample luminal antigens and subsequently migrate to lymph nodes for the initiation of adaptive T and B cell responses [113, 114]. These approaches provide the structural basis for efficient antigen delivery across the epithelium, yet durable immune protection depends on the use of appropriate adjuvant strategies.

## 5. Adjuvant Strategies for Enhancing Mucosal Vaccination

To enhance the efficacy of oral and intranasal mucosal vaccines, the use of adjuvants is necessary to strengthen antigen recognition, promote immune activation, and overcome the physiological barriers presented by mucosal tissues. Adjuvants are generally classified into two categories: synthetic molecular immunostimulants and natural compound-based enhancers. Both categories modulate innate and adaptive immunity, influence T cell polarization, and contribute to the establishment of long-term mucosal immune memory. Concurrently, adjuvant strategies are critical for enhancing vaccine efficacy, as they function to balance Th1/Th2 responses, promote sIgA production, support T cell activation, and strengthen mucosal barrier function.

Among synthetic adjuvants, CpG-oligodeoxynucleotides (CpG-ODNs) and IFN lambda 1 (IFN-*λ*1) are particularly promising. A recent study reported that nasal delivery of CpG-ODN-loaded cationic liposomes induces IL-6 secretion in nasal tissues, which is essential for antigen-specific IgA production but has minimal influence on systemic IgG levels [115]. Similarly, TLR9 upregulation observed during PEDV infection was found to be associated with increased intestinal IgA responses, highlighting the role of TLR9 signaling in mucosal immunity [116]. IFN-*λ*1, by enhancing antiviral defenses in epithelial cells, also supports the generation of durable immune memory through its cooperation with local T and B cell responses, making it a promising adjuvant candidate for both enteric and respiratory mucosal vaccines [117]. Beyond synthetic molecules, increasing attention has been directed toward natural immunomodulators.

Natural compound-based enhancers have also received considerable attention because of their favorable safety profile and immunomodulatory properties. Lactoferrin promotes DCs maturation, increases IL-6 and B cell-activating factor (BAFF) expression, and supports IgA class switching and secretion [118, 119]. In addition to its immunomodulatory properties, lactoferrin blocks the binding of the PEDV S protein to heparan sulfate proteoglycans, thereby combining antiviral activity with immune enhancement at mucosal sites [120]. Similarly, astragalus polysaccharides (APS) have been shown to increase milk and mucosal IgA titers in vaccinated sows, thereby enhancing passive immunity in piglets [121]. Moreover, GSLS, when administered orally prior to parenteral vaccination, enhance DC phagocytosis, promote CD4^+^ T cell activation, and increase PEDV-specific intestinal IgA production in mice [122]. Furthermore, when supplemented in piglet diets at a dosage of 0.1%, synbiotics improve intestinal barrier function, restore gut homeostasis, and reduce PEDV-induced inflammation, thereby conferring both immunological and physiological benefits [123]. Both synthetic molecular immunostimulants and natural compound-based enhancers play integral roles in strengthening mucosal immunity and optimizing vaccine efficacy.

## 6. Challenges and Future Prospects

Mucosal vaccines are widely regarded as a promising strategy for the control of SeCoVs infections because they can elicit sIgA and provide immune protection at the primary sites of viral entry. Nevertheless, several major obstacles hinder their development. At the antigen level, the rapid evolution of SeCoVs, particularly PEDV and PDCoV, frequently generates mutations within neutralizing epitopes of the S protein. Such antigenic variability reduces compatibility between vaccine strains and circulating viruses, thereby limiting cross-protective efficacy. At the delivery level, vaccine formulations must overcome enzymatic degradation, gastric acidity, and bile salts, while retaining sufficient stability to cross mucosal barriers and facilitate uptake by APCs, such as M cells and DCs [124–126]. The efficiency of delivery platforms is strongly influenced by physicochemical properties of the carriers, including NP size, surface charge, and ligand density, which require precise optimization to enhance mucosal transport and antigen presentation [127]. In addition, many delivery platforms display genetic instability, high sensitivity to gastrointestinal conditions, and strict cold-chain requirements, all of which complicate large-scale production and deployment [128]. Finally, some live bacterial vectors are capable of inducing strong mucosal immune responses, but their development is limited by biosafety concerns, including uncontrolled colonization and horizontal transfer of antibiotic resistance genes [129].

Progress in SeCoVs mucosal vaccine development is expected to result from advances in antigen design, delivery strategies, and evaluation models. At the antigen level, the incorporation of conserved epitopes together with suitable adjuvants is anticipated to broaden the spectrum of protection and extend immune durability. In addition, the optimization of delivery platforms can enhance antigen stability, facilitate trans-epithelial transport, and improve immune activation through multiple cellular pathways. Moreover, the establishment of predictive and standardized evaluation tools will be essential for guiding rational vaccine design. Recent technological innovations, including intestinal organoids, gut-on-chip systems, single-cell RNA sequencing, and spatial transcriptomics, provide unprecedented opportunities to investigate mucosal immune architecture and characterize vaccine-induced responses with high precision [130]. Together, these strategies are expected to advance the understanding of mucosal immunity and accelerate the development of effective and durable vaccines against SeCoVs.

In conclusion, oral and intranasal mucosal vaccines represent a promising strategy for controlling SeCoVs by establishing immune protection at mucosal surfaces. Their success depends not only on delivery platforms that can protect antigens from degradation and direct them to inductive sites, such as M cells and DCs, but also on mechanisms that can effectively activate immune responses. Effective mucosal immunity activation requires the induction of sIgA, T_RM_, and GC B cell responses, which collectively restrict viral replication, block transmission, and provide durable protection. In addition, the incorporation of adjuvants can further enhance the immune activation. Looking forward, the integration of optimized delivery systems with rational immune activation strategies will be critical to the development of safe, effective, and broadly protective mucosal vaccines against SeCoVs.

## Figures and Tables

**Table 1:** Comparison of structural features, immune activation mechanisms, and research applications of oral and intranasal mucosal vaccine delivery platforms.

Platform type	Structural features	Antigen protection and delivery mechanism	Type of immune activation	Advantages	Representative applications
Nanoparticles	Tunable particle size, surface modifiability, and favorable biocompatibility	Encapsulate antigens to prevent gastric and enzymatic degradation; enhance M cell and DC uptake through small particle size; enable surface functionalization with specific targeting ligands	Induce mucosal sIgA production; balance Th1/Th2 responses; promote GC reactions and TRM formation	Provide tunable structures for co-delivery of antigens and adjuvants; allow sustained antigen release and targeted delivery	PLGA-GSLS formulation enhances PEDV mucosal immunity [55]; LNPs deliver PEDV NTD [56]; intranasal mRNA-LNPs induce local IgA responses [57]
Hydrogels	Microporous structure with high hydration capacity; chemically or physically cross-linked network; tunable softness, strength, and environmental responsiveness	Protect antigens from enzymatic degradation; enable slow and sustained antigen release; enhance immune-cell uptake and activation; reinforce mucosal immune responses	Induce strong IgA and IgG production; activate DCs; promote T_RM_ and CD8^+^ T cell responses	Exhibit excellent mucosal compatibility; provide prolonged antigen exposure; facilitate enhanced uptake in NALT and PPs	Calcium-alginate hydrogel enhances PEDV mucosal immunity [58]
Engineered probiotics	Genetically modified probiotic strains engineered to express or secrete heterologous antigens; capable of displaying immune ligands on the bacterial surface and colonizing mucosal sites for continuous antigen delivery	Target antigen delivery to M cells and DCs; targeted M cell/DC delivery; present immune ligands on the surface to enhance mucosal signaling	Induce sIgA production; Th2-biased CD4^+^ T-cell activation; establish durable mucosal immune memory	Provide GRAS-level biosafety; enable natural colonization at mucosal sites; support continuous in situ antigen expression and immune stimulation	*Lactobacillus* expressing PEDV COE enhances intestinal IgA [59]; CRISPR-edited *L. casei* (VP4) [60]; multi-antigen probiotic vaccines confer protection against multiple pathogens [61]
Recombinant viral vectors	Genetically engineered viral vectors designed to deliver and express target antigens in host cells	Protect antigens within the viral genome; deliver them to host cells through infection; enable antigen expression and presentation via MHC pathways	Induce strong Th1 and CTL responses; elicit high levels of neutralizing IgA and IgG antibodies	Mimic natural infection; achieve high transduction efficiency; stimulate potent and balanced humoral and cellular immune responses	rAd5-S induces IgA and IgG responses in pigs [62]; rAd5-S1N reduces viral shedding [63]; intranasal PanAd3–MVA activates nasal T_RM_ responses [64]

## Data Availability

No new data were generated or analyzed in support of this review. All the data cited in this article are available from the referenced publications.
